# An Unusual Presentation of a Patient with Leg Ulcers: A Case Report

**DOI:** 10.7759/cureus.6293

**Published:** 2019-12-05

**Authors:** Sana Zafar, Khurram Saleem, Aqeela Rashid

**Affiliations:** 1 Internal Medicine, University College of Medicine, University of Lahore, Lahore, PAK; 2 Internal Medicine, Jinnah Hospital, Allama Iqbal Medical College, Lahore, PAK

**Keywords:** leg ulcers, anemia, thalassemia major, non transfusion dependent

## Abstract

Leg ulcers have been a common presentation in clinics; disruptions in the mechanism of ulcer healing are vascular insufficiency, anemia, metabolic disturbances, neuropathy, and autoimmunity. The term 'non-transfusion-dependent thalassemia' encompasses the milder forms of thalassemia traits that require intermittent or no transfusion at all, and are mostly associated with leg ulcers.

We present the case of a 19-year-old female with beta-thalassemia major who presented with non-healing leg ulcers and anemia. The clinical findings and lab evidence suggested hemolytic anemia evidenced by pathologic fractures, hepato-splenomegaly, and normal iron studies. Hemoglobin electrophoresis confirmed beta-thalassemia major with its complications including adrenal insufficiency and pathological fractures, all of which remained well compensated till the second decade of life.

## Introduction

Chronic lower limb ulceration is a frequently encountered presentation in clinical settings, with significant morbidity and psycho-social impact. affects about 1% of the adult population and 3.6% of people older than 65 years [1]. The causes of leg ulcers are multifactorial, the most common being venous insufficiency, arterial insufficiency, neuropathy, and diabetes. Indolent, non-healing ulcers are a feature of sickle cell disease, thalassemia, and other hemolytic anemias. Disruption of microvascular circulation is the underlying mechanism in such cases. Thrombotic and occlusive disease, for instance, antiphospholipid antibody syndrome, protein C and protein S deficiency, and cryoglobulinaemia also cause skin necrosis leading to ulceration and gangrene [2]. Here we present an unusual case of a young female with non-healing leg ulcers and anemia.

## Case presentation

A 19-year-old female presented with a history of exertional shortness of breath, palpitations, dizziness for one and a half years, which had gradually worsened over a period of one month. The associated symptoms included left-sided upper abdominal heaviness, early satiety, easy bruising, joint pains, oral ulcers, and bone pains. She presented with recurrent ulcers on the legs and ankle requiring multiple antibiotics and visits to surgical clinics for the past three years. Her parents had a consanguineous marriage; in the past, she also had a spontaneous/pathological fracture of the right femur, which was treated but no medical record was available. There was no history of fever, significant blood loss, blood transfusions, lead exposure, early morning dark colored urine, loose stools, vomiting, abdominal pain, lumps and bumps, or prolonged history of drug intake.

**Figure 1:**
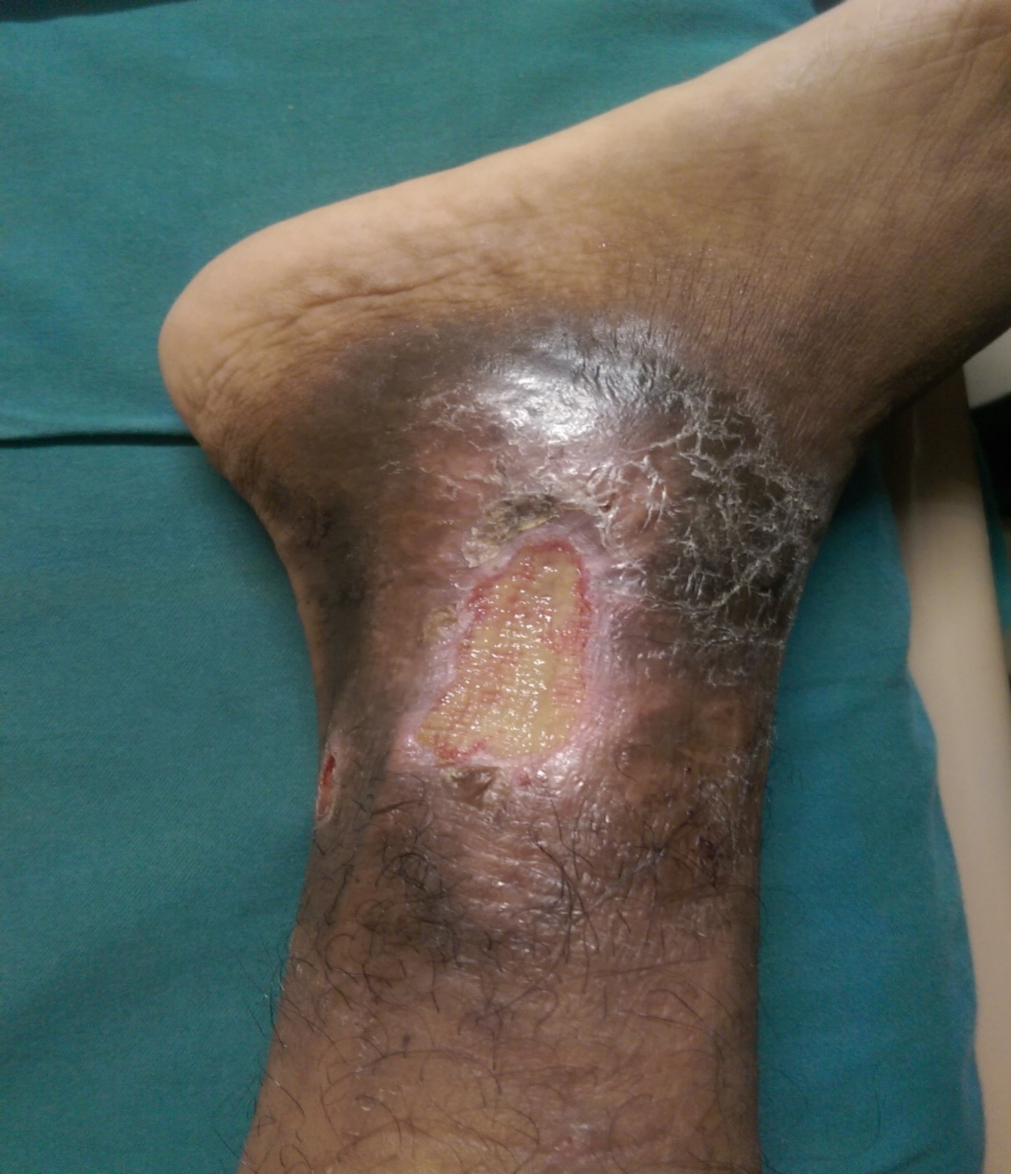
Leg ulcer (non healing)

Examination findings were as follows: pallor, hepato-splenomegaly, no lymphadenopathy. There were short systolic flow murmurs heard along the left sternal edge. In the lower limbs, the right leg showed a healed ulcer on the lateral malleolus. On the left leg, there was an approximately 10 cm elongated ulcer with undermined edges and pus at the base, along with surrounding skin excoriation and lichenification (Figure 1).

Initial investigations were done as shown in Table 1. The results showed microcytic hypochromic anemia with normal iron studies.

**Table 1:** Initial investigations showing microcytic hypochromic anemia

	2018	Reference Ranges
Hemoglobin (g/dl)	7.1	12-16
Total leucocyte count (*10^9^/L)	9.2	4-11
Platelets (*10^9^/L)	575	150-450
Mean corpuscular volume (fL)	61	85-95
Mean corpuscular hemoglobin (pg)	22	27-31
Mean Corpuscular hemoglobin concentration (pg/dl)	32	32-38
Peripheral blood smear	Microcytic hypochromic RBCs, anisopoikilocytosis, tear drop cells, target cells	
Reticulocytes (%)	2.5	0.2-2.0
Lactate dehydrogenase (u/l)	317	Upto 250
Iron (µg/dl)	300	65-175
Ferritin (ng/ml)	736.6	Upto 280
Total Iron binding capacity (µg/dl)	286	250-400
Vitamin B12 (pg/ml)	260	160-914
Red cell folate (ng/ml)	750	529-2322
Bilirubin (mg/dl)	2.3	0-1.2
Alanine aminotransferase (U/L)	23	Up to 32
Aspartate aminotransferase (U/L)	42	Up to 32
Alkaline phosphatase (U/L)	61	35-106

For the workup of the leg ulcer, she underwent an incisional biopsy of the leg ulcer which showed chronic granulomatous inflammation with no evidence of malignancy; periodic acid-Schiff (PAS) stain was negative for fungus. Lower gastrointestinal (GI) endoscopy was done for the possibility of inflammatory bowel disease but colonoscopy showed normal mucosa with normal haustration pattern. Doppler studies of the lower limbs showed normal arterial and venous flow. Magnetic resonance imaging (MRI) of the left leg showed multiple hyper-intense signals in the tibia, calcaneus and talus with no evidence of osteomyelitis. Furthermore, auto-immune workup and vasculitis screen was done as shown in Table 2.

**Table 2:** Autoimmune workup Anti-dsDNA: anti-double-stranded deoxyribonucleic acid; RA factor: rheumatoid factor; ANCA: antineutrophil cytoplasmic antibodies.

	Patient’s Value	Reference Range
ANA	Nucleolar pattern 1:100	< 1:100
anti-dsDNA (IU/ml)	83	>25 (positive)
RA factor (IU/ml)	<20.0	>50.0 (positive)
Complement C3 (mg/dL)	123	83-193
C4 (mg/dL)	30.1	15-57
c-ANCA	0.62	>1.10 (positive)
p-ANCA	0.78	>1.10 (positive)

She was prescribed oral steroids by the dermatology team considering the diagnosis of ANCA negative vasculitis (leucocytoclastic angiitis) which showed no improvement during her hospital admission. Hemoglobin electrophoresis was done and it supported her clinical condition to reach the final diagnosis of thalassemia major. The results are shown below (Table 3).

**Table 3:** Hemoglobin electrophoresis HBA: hemoglobin A; HBF: hemoglobin F.

	Patient’s value	Reference range
HBA	12%	96.5-99.5%
HBA_2_	1.9%	0-3.5%
HBF	86.1%	<2% (age dependent)

The patient was diagnosed as a case of beta-thalassemia major, which remained non-transfusion dependent up to the second decade of life. Radiographic findings suggestive of marrow cavity expansion were present as well. Another finding was a significant postural drop of blood pressure for which serum cortisol and adrenocorticotropic hormone (ACTH) levels were done, which were suggestive of adrenal insufficiency (Table 4).

**Table 4:** Lab values suggesting adrenal insufficiency ACTH: adrenocorticotropic hormone.

	Patient’s value	Reference range
Cortisol (9 AM) µg/dL	2.3	3.7-19.4
ACTH (pg/ml)	68.4	9-52

**Figure 2:**
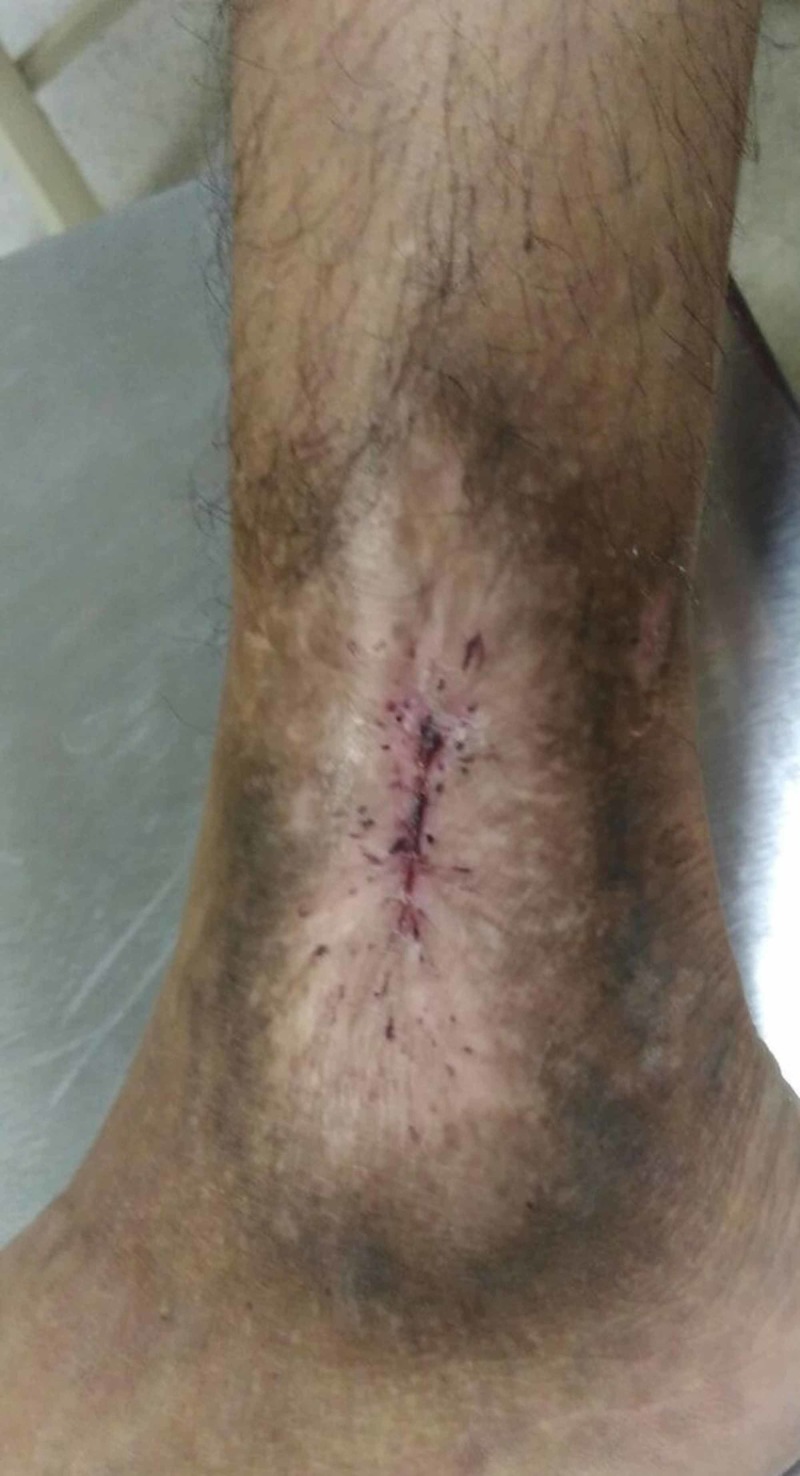
Healed leg ulcer

The patient was transfused with packed red blood cells and hemoglobin was built up to 10 g/dL along with ciprofloxacin, 500 mg twice daily, for treatment of secondary wound infection. This resulted in complete wound healing (Figure 2).

Also, daily replacement of oral steroids (prednisolone 5 mg + 2.5 mg) was given, which improved her symptoms of postural dizziness as noted on a follow-up visit. Oral iron chelation therapy, deferasirox was given to manage iron overload associated with thalssemia-related hemolysis and blood transfusions. 

## Discussion

**Figure 3:**
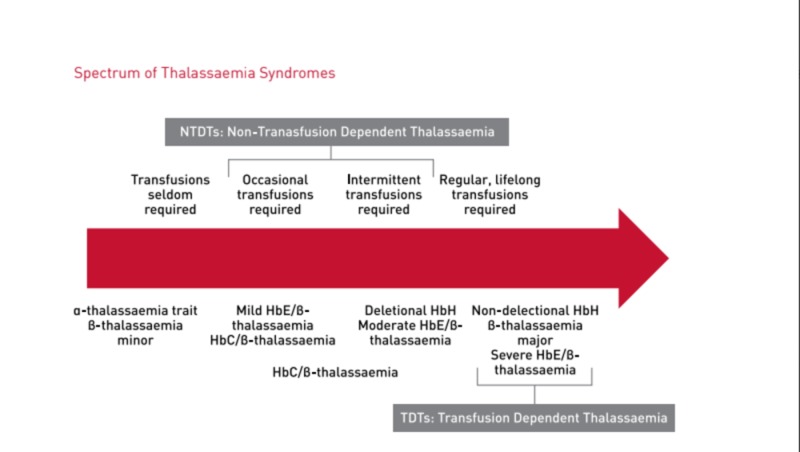
Spectrum of thalassemia syndromes Image courtesy: Guidelines for the Management of Transfusion Dependent Thalassaemia (TDT) [4].

Inherited hemoglobin disorders have been divided on the basis of defective chain synthesis (α- and β-thalassemia) and structural variations (Hemoglobin S, C, and E). Various clinical presentations result from either simultaneous inheritance of two different mutations or their co-existence with structural variants. However, in all the cases, the result is defective red blood cell production, with peripheral hemolysis and anemia [3]. For this reason, transfusion dependence has been one of the essential factor in distinguishing the various thalassemia phenotypes and their severity. Figure 3 illustrates the different thalassemia traits according to the transfusion dependence.

Patients with transfusion-dependent thalassemia (TDTs) require regular blood transfusion to survive. β-thalassaemia major, severe HbE/β-thalassemia, non-deletional hemoglobin H (HbH) disease are included in this category. Non-transfusion-dependent thalassemia includes β-thalassemia intermedia, hemoglobin E (HbE)/β-thalassemia, and HbH disease. This sub-group require seldom or intermittent transfusion according to the clinical stress [4].

**Figure 4:**
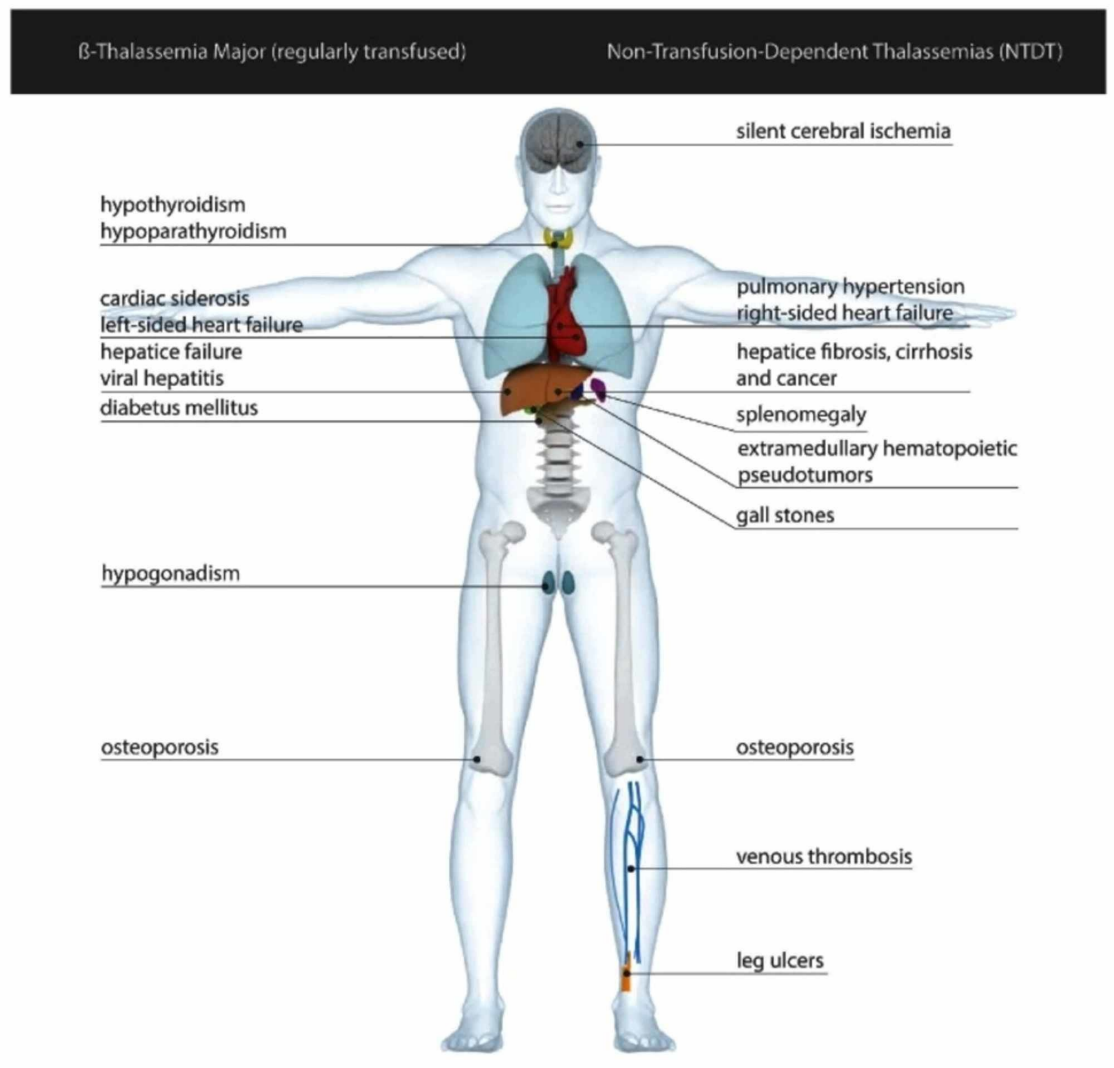
Complications of transfusion-dependent and non-transfusion-dependent thalassemia major Image courtesy: Non-transfusion-dependent thalassemias [3].

With increasing age, the complications associated with thalassemia syndromes develop. These may be related to the transfusion dependency or a part of the disease itself. These include secondary hemosiderosis, endocrine abnormalities, bone disease, pathological fractures, and hemodynamic changes. The development of pulmonary hypertension and cardiac arrhythmias can be fatal. Adequate physician education and understanding is required to preempt the complications and diagnose them earlier in the course of the disease. Figure 4 represents the commonly encountered complications of thalassemia as per their transfusion dependence [5].

The association of leg ulcers has been observed more in patients of non-transfusion dependent thalassemia, especially in the elderly. This is because of the increased skin and vascular fragility in the elderly, due to reduced oxygen supply at the peripheries. Consequently, they have an increased risk of developing leg ulcers after minimal trauma [6]. Local iron deposition is also considered to be a contributing factor in poor healing of leg ulcers especially when heme component of the red blood cells deposits locally giving a dark hue. Thus higher rates of leg ulcers have been reported in patients with a non-transfusion-dependent spectrum of the disease having iron overload [6].

However, our patient's case was an unusual presentation of beta-thalassemia major, who remained well-compensated up till the second decade of life without any blood transfusions. Rather she presented with complications of the disease in the form of leg ulcers, adreno-cortical insufficiency and pathological fractures.

## Conclusions

Thalassemia is a hemoglobinopathy with a spectrum of clinical presentations. Although thalassemia major presents earlier in life with resultant dependence on blood transfusion in the first few years of life, there can be a variability in the presentation, as in our patient who presented with recurrent leg ulcers and features of anemia and remained well-compensated until the adolescent age when the need for regular transfusions arise.
